# Primary isolated hydatid cyst of the spleen: A case report

**DOI:** 10.1016/j.ijscr.2024.109552

**Published:** 2024-03-16

**Authors:** Maissa Jallali, Mohamed Ali Chaouch, Hanen Zenati, Hiba Ben Hassine, Jamel Saad, Faouzi Noomen

**Affiliations:** a Department of Visceral and Digestive Surgery, Monastir University Hospital, Monastir, Tunisia; b Department of radiology, Monastir University Hospital, Monastir, Tunisia

**Keywords:** Echinococcosis, Splenic cyst, Primary splenic hydatidosis, Surgery

## Abstract

**Introduction and importance:**

Primary (isolated) splenic hydatid cyst is rare and accounts for less than 2 % of hydatid patients, even in endemic regions. Diagnosis of splenic hydatid cyst can be challenging due to the rarity of the condition and its nonspecific symptoms. Surgery is the mainstay of treatment. This case report discusses management options for such a rare condition.

**Case presentation:**

We present a 33-year-old female patient with abdominal pain for six months and splenomegaly. Ultrasonography and CT scan showed a giant splenic cyst with clear walls and multi-vesicular contents suggestive of a hydatid cyst. There was no involvement of the liver or other organs. Indirect hemagglutination was positive for Echinococcus. Through a left subcostal incision total splenectomy was performed. The patient was discharged from hospital on the sixth postoperative day. No local recurrence was detected during postoperative follow up.

**Case discussion:**

Primary splenic hydatid disease is rare. It may be detected incidentally or present with nonspecific complaints. If untreated, a splenic hydatid cyst can lead to various potentially severe complications, including cyst rupture and secondary infection. Standard treatment is open total or partial splenectomy: preservation surgery should always be considered, to avoid post splenectomy infection, especially in young patients.

**Conclusion:**

Primary splenic hydatid cyst is rare even in endemic areas. Symptoms may be non-specific. Standard treatment is open total or partial splenectomy.

## Highlights

• The spleen is the third most common site of hydatid disease, after the liver and lungs
• Splenic hydatid cyst can be either primary (isolated to the spleen only) or secondary (accompanied by hydatid cysts in other organs)
• Primary splenic hydatid cyst is rare even in endemic regions
• Diagnosing hydatid cyst disease of the spleen can be challenging due to the rarity of the condition and often nonspecific symptoms
• Surgery is the usual management of hydatid cyst of the spleen

## 1 Introduction

Hydatid disease (HD) is a parasitic infection caused by Echinococcus granulosus. The parasite primarily spreads through contact with infected animals, such as dogs or livestock, and its larvae can form cysts in different tissues [1]. HD disease can affect any organ in the human body. The most common site of disease is the liver (50–70 %), followed by the lungs (25 %) [2]. The spleen hydatid disease (SHD) is a rare clinical and the frequency is reported to be 0.5 to 4 % of abdominal hydatid diseases [3]. Diagnosing SHD can be challenging due to their nonspecific symptoms and the rarity of the condition. If left untreated, SHD can lead to various complications, including cyst rupture and secondary infection. This case report was carried out according to the recent SCARE criteria [4].

## 2 Case presentation

**Fig. 1:**
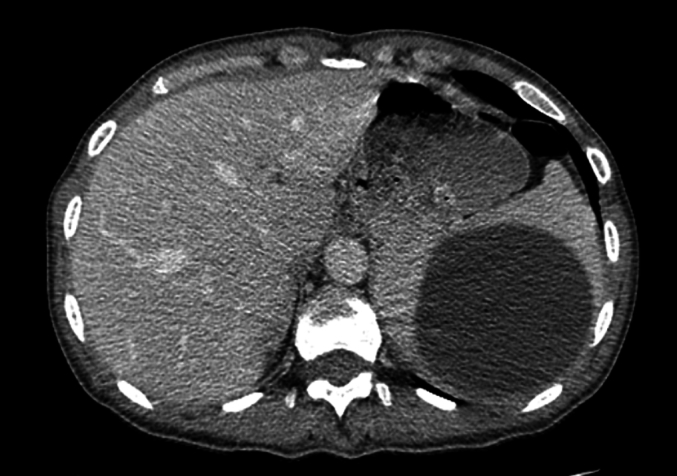
CT axial scan view appearance of a primary splenic hydatid cyst.

**Fig. 2:**
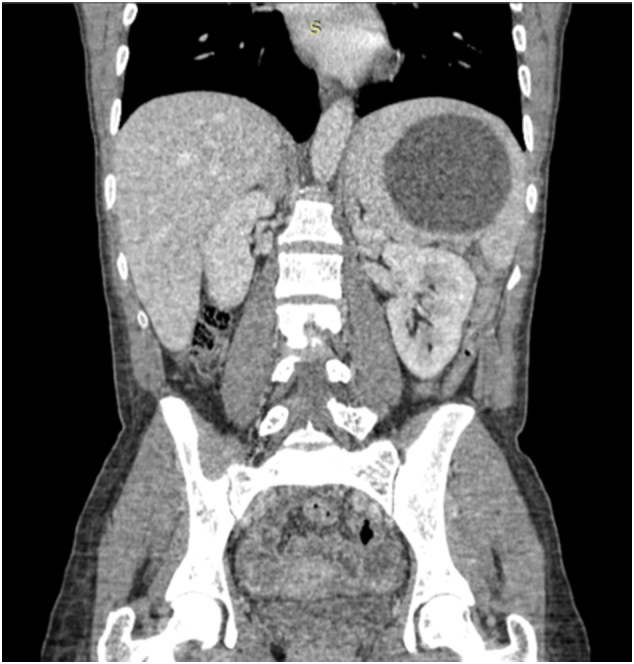
CT coronal view of a primary splenic hydatid cyst.

A 33-year-old woman patient, without medical our surgical history, was referred to our general surgery department, due to heaviness-type abdominal and non-radiating lasting for six months. The patient was hemodynamically stable. It was noted to have a temperature of 37, a blood pressure of 120/60 mm/Hg, a heart rate of 70/min and a respiratory rate of 18/min. In the examination, we noticed a left upper quadrant tenderness and splenomegaly, there was no history of pet dogs or sheep at home. All the routine laboratory tests of the patient were unremarkable. Ultrasonography (USG) of the abdomen was performed that showed a splenic cyst with a clear wall and multivesicular contents suggestive of a hydatid cyst of the spleen measuring 12 cm*9 cm. CT abdomen confirmed a cystic lesion of the giant spleen (Fig. 1). There was no involvement of liver or other organs. Indirect hemagglutination (HAI)was positive for Echinococcus. The patient was from an endemic country of hydatid disease and she consumed undercooked meats. The diagnosis of hydatid cyst was retained. Laparotomy was performed through the left subcostal incision. Surgical exploration revealed a hydatid cyst occupying the upper pole of the spleen (Fig. 2). Total splenectomy was realized. Macroscopic and microscopic examination of the specimen confirmed Hydatid cyst. The patient was discharged from hospital on the sixth postoperative. She was vaccinated against pneumococcus and haemophilus. No local recurrence was detected after one year of follow up.

## 3 Discussion

Hydatid disease is endemic in North Africa, the Middle East, Australia, and South America [5]. It can be found in any organ and mostly affects the liver and lungs [2]. Sympathetic hydatid cystis is the third abdominal location and can be primary, isolated from the spleen only or secondary accompanied by hydatid cysts in other organs. Primary splenic hydatid cyst is rare and represents less than 2 % of patients, even in endemic regions [6,7]. Symptoms of splenic hydatid cysts can vary depending on the size and location of the cyst. SHC is characterised by clinical latency due to its slow growth, and diagnosis is often incidental during radiographic investigations for other reasons [8,9]. The clinical symptoms of SHC are nonspecific symptoms such as pain, fullness, discomfort, and the presence of a mass in the left upper quadrant mass. Enlarged SHCs can, in rare cases, reveal their presence through complications [8]. Complications of SHC include infection, fistulization to adjacent organs, intra-abdominal rupture leading to anaphylactic reactions or hydatidosis, renal arterial compression causing systemic hypertension, and splenic vein compression resulting in segmental portal hypertension [9,10]. Physical examination reveals nothing except rare cases of painless splenomegaly. The diagnosis of splenic hydatid cysts (SHC) remains a challenge due to often asymptomatic or non-specific symptoms. The differential diagnosis of SHC includes epidermoid cysts, large solitary abscess or hematoma, cystic hemangiomas, intrasplenic pancreatic pseudocyst, and cystic neoplasm of the spleen (lymphangiomas). The combination of hydatid serology and imaging is contributive for SHC diagnosing in almost 90 % of cases [10]. Ultrasound remains the initial imaging modality of choice for splenic hydatid cysts. In fact, Gharbi et al. allowed for a standardised classification following the natural history of the parasite [11]. CT scans has superior specificity (95–100 % versus 80–90 %). It allows us to precisely determine the number, size, anatomical location of cysts, and anatomical contacts with surrounding vessels and organs, as well as detect complications such as infection [12]. Parietal calcifications, the ‘floating membrane’ sign, echinogenic daughter cysts, and concomitant extra-splenic cystic lesions represent key imaging features significantly impacting the diagnostic confidence for splenic hydatid cysts. Magnetic resonance imaging has some advantages in terms of providing cystic pathological characteristics [13]. Serological tests are used in diagnosis of hydatid disease and follow-up of recurrent cases. Indirect hemagglutination and enzyme-linked immunosorbent assay (ELISA) are frequently employed due to their increased sensitivity and specificity, respectively [14].

Many management options for SHC have been described in the literature, including surgery, percutaneous management, drug therapy, and the observation stage [15]. However, mainly treatment of hydatid spleen cysts remains surgical for avoiding spontaneous or traumatic rupture. Standard surgical management of SHC is cyst enucleation, total or partial splenectomy [16]. Spleen preservation, by realising cyst enucleation or partial splenectomy, in SHC surgery minimises long-term immune risks but presents an increased risk of bleeding when incising the splenic tissue, and unroofing the cyst wall had a risk of severe postoperative infection due to the residual space. Therefore, in the case of multiple splenic cysts or a large cyst, total splenectomy is advised, especially when there are adhesions between the spleen and nearby organs. Laparoscopic splenectomy is being increasingly performed at advanced laparoscopic centres. However, some authors believe it is unsafe due to the risk of anaphylactic shock and intra-peritoneal dissemination. Spillage of protoscolex-rich fluid during surgery occurs in 5 %–10 % of cases [17].

## 4 Conclusion

Primary splenic HC is rare even in an endemic area. The clinical symptoms are non-specific. The abdomen and CT scan are helpful in making the diagnosis. Management must be individualised for each patient. In our patient, total splenectomy was the best choice to avoid complications and recurrence.

## Patient consent

Written informed consent was obtained from the patient to publish this case report and accompanying images. On request, a copy of the written consent is available for review by the Editor-in-Chief of this journal.

## Ethical approval

Ethical approval is exempt/waived at our institution.

## Funding

No funding.

## Author contribution

All the authors participated in the manuscript and validated the final version of the manuscript

## Guarantor

Mohamed Ali Chaouch.

## Research registration number

Not applicable.

## Declaration of competing interest

The authors declare no conflict of interest.
